# The association between relative fat mass (RFM) and lumbar bone density in US adults: Insight from 2011–2018 NHANES

**DOI:** 10.1371/journal.pone.0323243

**Published:** 2025-05-08

**Authors:** Hanwen Zhang, Jian Mei

**Affiliations:** 1 Department of Plastic, Hand and Reconstructive Surgery, University Hospital Regensburg, Regensburg, Germany; 2 University of Regensburg, Regensburg, Germany; The University of Sydney School of Medicine, AUSTRALIA

## Abstract

**Background:**

The relationship between obesity and osteoporosis has been widely studied. Studies have shown that relative fat mass (RFM) can be used to predict obesity. However, its relationship with bone mineral density (BMD) is unclear. The aim of this study was to investigate the relationship between RFM and lumbar BMD.

**Methods:**

We used data from the National Health and Nutrition Examination Survey (NHANES) from 2011 to 2018. Multiple linear regression models were applied to examine the relationship between RFM and lumbar BMD. We used smoothed curve fitting and threshold effect analysis to check for non-linear trends. Subgroup analyses were performed to explore differences in various populations. The Receiver operating characteristic (ROC) curve was used to evaluate the diagnostic potential of body mass index (BMI), weight-adjusted waist circumference index (WWI), waist circumference (WC), and RFM for OP.

**Results:**

A total of 10636 participants were included. Multiple linear regression showed a significant negative link between RFM and lumbar BMD. In subgroup studies and smoothed curve fitting, RFM was consistently associated with lower BMD across subgroups. ROC curve indicate that RFM is more sensitive than BMI, WWI and WC in diagnosing OP capacity.

**Conclusion:**

RFM is negatively associated with lumbar BMD in US adults. This suggests that RFM contributes to the study of low BMD.

## 1. Introduction

Osteoporosis (OP) is a metabolic bone disease that causes bone loss and microstructure damage [1]. Osteoporosis is typically asymptomatic, but its consequences include fragility fractures, which can lead to pain, scoliosis, and other deformities. In recent years, fractures due to OP have become more common as the population ages. This seriously affects the quality of life of elderly patients [2]. In the United States, the cost of OP prevention and treatment reaches up to $17 billion per year [3]. However, OP is influenced by genetics, age, lifestyle, and other diseases, making prevention and diagnosis difficult.

Obesity is also a growing global health concern. It is associated with a variety of health problems, including chronic inflammation and systemic diseases [4]. Obesity has been shown to be potentially linked to low BMD [5]. Traditionally, obesity is usually diagnosed using BMI. Although utilizing BMI to predict OP is contentious, several studies have revealed a strong association between BMI and BMD [6,7]. It has also been stated that BMI cannot distinguish between body form, fat, and muscle [8]. To improve obesity assessment, researchers have used waist circumference (WC) as a measure of central obesity [9]. The strong association between WC and BMI makes it challenging to use WC as an independent obesity criterion [10,11]. As a result, some scholars found that the weight-adjusted waist circumference index (WWI) could effectively combine weight and waist circumference [12]. Wang *et al*. found that WWI was negatively correlated with total BMD in adolescents [13]. Recently, it has been suggested that RFM is more accurate than BMI in assessing obesity capacity [14]. RFM is effective in predicting visceral obesity using a combined assessment of WC and height to assess fat mass [15]. Some researchers have suggested that RFM can help predict various diseases [16–18]. However, there are few studies on RFM and BMD. Therefore, we used NHANES data to find out the association between RFM and lumbar BMD. We hope to find a stable index for the assessment of lumbar BMD.

## 2. Methods

### 2.1 Information population

**Fig 1:**
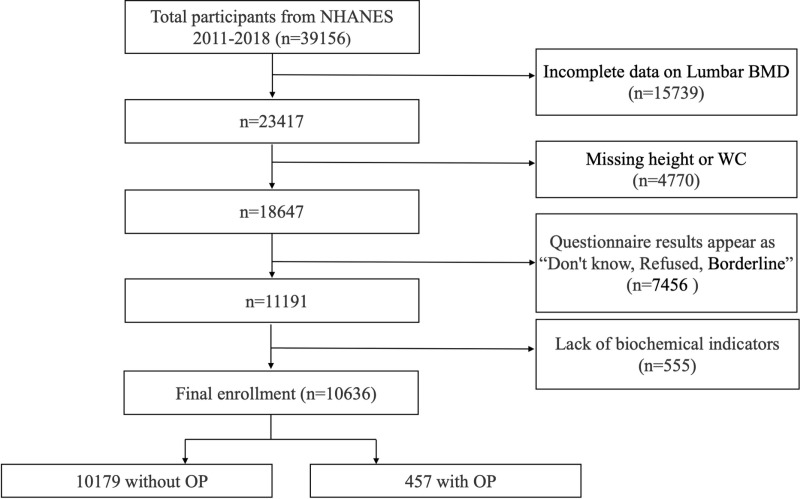
Flowchart of NHANES sample selection 2011–2018.

NHANES collects a wide range of health and nutrition data with the goal of assessing health and nutrition status, as well as understanding and tracking national health trends. Our studies are based on NHANES database data from 2011–2018. We excluded, in order to lower error and improve data availability, 15739 participants with missing lumbar BMD or age < 20 years; 4770 participants with missing height or waist circumference results; 7456 participants with direct exclusions such “don’t know, refused, or borderline” on questionnaire results; and 555 participants with missing biochemical indicators. At last, 10636 people were counted. The full process is illustrated in Fig 1. The studies involving human participants were reviewed and approved by NCHS Research Ethics Review Board. Written informed consent for participation was not required for this study in accordance with the national legislation and the institutional requirements.

### 2.2 RFM

The calculation of RFM is as follows: male RFM = 64 - (20 × waist/height); female RFM = 76 - (20 × waist/height) [15]. Health technicians were trained to obtain all body measurements at a mobile examination center (MEC). This was accomplished in collaboration with health technicians and recorders, and it was reviewed by an expert examiner to illustrate the appropriate application of each measurement. Post-recorded heights and weights are reviewed to ensure that the entire body measurement record is sound. In cases of extreme shortness or extra height, overweight, and underweight, analysts reviewed the distribution of body measurements and considered whether to include them in the study.

### 2.3 Lumbar BMD

Dual-energy X-ray absorptiometry (DXA) is a widely used method in BMD testing due to its minimal radiation exposure, ease of use, and speed. Lumbar BMD is the bone density of L1-L4 and is typically measured using DXA. The Apex version 5.6.0.5 software was employed to acquire spine radiographs using the Hologic Discovery A densitometer. In order to guarantee the reliability of data and the safety of the test, the data collection contractor’s personnel conducted measurements in conjunction with monitoring technicians through field observations. Diagnostic criteria for OP were based on the mean peak BMD and corresponding standard deviation (SD) of white females aged 20–29 years in the NHANES III database [19,20]. Subsequently, the population was categorised into OP (T-value ≤ -2.5), osteopenia (-2.5 < T-value ≤ -1) and normal (and T-value > -1) using established methods for converting BMD levels in the lumbar spine to T-values [21]. Where osteopenia and normal bone mass were considered as no osteoporosis.

### 2.4 Covariates

Given the findings of prior research on the correlation between adiposity and BMD [22,23], we incorporated a diverse array of covariates that could potentially influence the results. Age, gender (male or female), race (Mexican American, other Hispanic, non-Hispanic white, non-Hispanic black, and other races), educational level (Less than 9th grade, 9th-11th grade, High school graduate/GED or equivalent, Some college or AA degree, College graduate or above), poverty-to-income ratio (PIR), total cholesterol (TC), high density lipoprotein (HDL-C), vitamin D, BMI, blood urea nitrogen, total calcium, WWI (WC divided by the square root of weight) and moderate recreational activities (whether they will participate in any moderately intense 1-week exercise, fitness, or recreational activities), hypertension (whether or not they have ever been informed by a doctor or other health professional that they have high blood pressure), diabetes (whether or not they have ever been informed by a doctor or other health professional that they have diabetes), and smoking (smoking more than 100 cigarettes in their lifetime).

### 2.5 Statistical analyses

The statistical analyses were adjusted based on the NHANES analytic criteria. Initially, we employed chi-square and t-test methods to analyze the demographic data by categorizing it into RFM quartiles. The quartiles were defined as follows: Quartile 1 (7.76–28.01), Quartile 2 (28.01–33.71)), Quartile 3 (33.71–41.75), and Quartile 4 (41.75–56.68). Continuous variables are represented as mean ± standard deviation (SD), while categorical variables are represented as percentages. Three models were developed to account for possible confounding factors: model 1 was not adjusted, model 2 was adjusted for age, gender, and race, and model 3 was adjusted for age, gender, race, educational level, PIR, TC, HDL-C, vitamin D, BMI, blood urea nitrogen, total calcium, moderate recreational activities, hypertension and diabetes, and smoking status. Data with more than 10% missing values were excluded, whereas data with less than 10% missing values for PIR, BMI, total calcium and total cholesterol were estimated using multiple imputation. Missing values were estimated using the proportional estimation method, replacing missing values with the corresponding median. In addition, subgroup analyses and interaction tests were conducted to examine the effects of gender, race, moderate exercise, hypertension, diabetes, and smoking. Nonlinearity was addressed using weighted generalised additive models and smoothed curve fitting. ROC curves were used to test the diagnostic ability of RFM, BMI and WC for OP. The DeLong test was used to test for statistical differences in ROC. The statistical analyses were conducted using R (version 4.2) or Empowerstats (version 2.0).

## 3. Results

### 3.1 Characteristics of the participant population

A total of 10636 adults took part in the study. There were 5433 men and 5203 women among them. The mean age (SD) was 39.23 (11.46) years. The group was made up of 3697 non-Hispanic whites (34.76%), 2301 non-Hispanic blacks (21.63%), 1654 Mexican Americans (14.7%), 1106 other Hispanics (10.4%), and 1968 people of other races (18.5%). The mean RFM (SD) for participants was 34.53 (8.94). People in the top quartile were more likely to be older, female, and Non-Hispanic White than people in the bottom quartile. People with higher RFM were also more likely to have a higher BMI, more schooling, and higher levels of TC and vitamin D (Table 1).

**Table 1:** Basic characteristics of participants.

	Q1 (7.76–28.01)	Q2 (28.01–33.71)	Q3 (33.71–41.75)	Q4 (41.75–56.68)	P value
Age (years, mean ± SD)	39.33 ± 11.64	39.14 ± 11.53	39.14 ± 11.53	39.64 ± 11.91	0.2386
Gender, (%)					0.0019
Male	49.33	51.45	54.55	52.22	
Female	50.67	51.45	45.45	47.78	
Race, (%)					0.0250
Mexican American	9.61	9.51	10.48	11.25	
Other Hispanic	7.05	7.14	7.36	7.55	
Non-Hispanic White	61.32	60.51	62.95	61.61	
Non-Hispanic Black	12.66	12.73	10.50	10.10	
Other Race	9.36	10.11	8.71	9.50	
Education level, (%)					0.0244
Less than 9th grade	3.14	3.41	3.73	5.10	
9-11th grade (Includes 12th grade with no diploma)	9.11	9.30	9.32	9.15	
High school graduate/GED or equivalent	21.19	21.48	21.90	21.93	
Some college or AA degree	34.91	32.65	31.74	32.54	
College graduate or above	31.64	33.16	33.32	31.27	
PIR (mean ± SD)	2.91 ± 1.63	2.89 ± 1.62	2.98 ± 1.62	2.87 ± 1.62	0.0622
Total TC (mmol/L, mean ± SD)	4.96 ± 1.07	4.91 ± 0.98	4.99 ± 1.02	4.95 ± 1.04	0.0398
HDL-C (mmol/L, mean ± SD)	1.39 ± 0.43	1.35 ± 0.40	1.36 ± 0.41	1.36 ± 0.41	0.0028
Vitamin D (nmol/L, mean ± SD)	61.21 ± 24.18	60.96 ± 24.04	63.17 ± 27.17	57.28 ± 25.12	<0.0001
BMI (kg/m^2^, mean ± SD)	24.06 ± 2.89	28.48 ± 4.77	28.74 ± 7.44	34.91 ± 6.28	<0.0001
WC (cm, mean ± SD)	86.49 ± 8.18	98.67 ± 13.70	96.83 ± 19.86	109.26 ± 13.53	<0.0001
WWI (cm/ √ kg)	12.94 ± 6.11	14.86 ± 7.17	14.26 ± 7.03	16.23 ± 7.53	<0.0001
Blood urea nitrogen (mmol/L, mean ± SD)	12.94 ± 6.11	14.86 ± 7.17	14.26 ± 7.03	16.23 ± 7.53	<0.0001
Total protein (g/L, mean ± SD)	72.47 ± 4.42	72.06 ± 4.43	71.57 ± 4.26	71.59 ± 4.29	<0.0001
Serum phosphorus (mg/dL, mean ± SD)	3.72 ± 0.57	3.70 ± 0.57	3.74 ± 0.59	3.70 ± 0.54	0.0326
Total calcium (mg/dL, mean ± SD)	9.47 ± 0.32	9.38 ± 0.32	9.33 ± 0.33	9.29 ± 0.36	<0.0001
Medium activities, (%)					<0.0001
Yes	48.33	44.70	47.13	41.93	
No	51.67	55.30	52.87	58.07	
Hypertension, (%)					<0.0001
Yes	13.46	25.01	19.64	34.06	
No	86.54	74.99	80.36	65.94	
Diabetes, (%)					<0.0001
Yes	3.32	6.98	6.45	13.13	
No	96.68	93.02	93.55	86.87	
Smoking, (%)					<0.0001
Yes	44.91	43.73	34.42	34.01	
No	55.09	56.27	65.58	65.99	
Lumbar BMD (g/cm^2^, mean ± SD)	1.08 ± 0.14	1.05 ± 0.14	1.02 ± 0.14	1.00 ± 0.16	<0.0001

### 3.2 Relationship between RFM and Lumbar BMD

**Fig 2:**
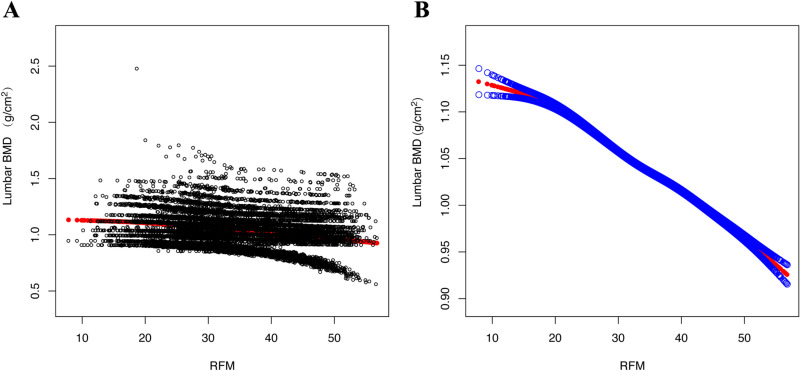
Nonlinear negative association between RFM and Lumbar BMD (g/cm^2^). **(A)** Each black dot represents a sample. **(B)** Solid red lines indicate smooth curve fits between variables. The blue band indicates the 95% confidence interval of the fit.

We found that RFM and lumbar BMD were negatively correlated (Table 2). For every unit increase in RFM in the top quartile (β= -0.079, 95% CI: -0.082, -0.076), lumbar BMD decreased by 0.079 g/cm^2^ in the fully adjusted model (P for trend <0.001). Using smooth curve fitting, we discovered a nonlinear negative relationship between RFM and Lumbar BMD (Fig 2). The saturation effect study found an inflection point K between RFM and lumbar spine bone mineral density. Each 1-unit increase in RFM was associated with a 0.002 g/cm^2^ decrease in BMD when K < 20.097, and a 0.005 g/cm^2^ decrease in BMD when K > 20.097 (Table 3).

**Table 2:** Association between RFM and Lumbar BMD.

	Model1	Model 2	Model 3
	β (95% CI)	β (95% CI)	β (95% CI)
RFM	-0.003 (-0.004, -0.003)	-0.003 (-0.004, -0.003)	-0.004 (-0.005, -0.004)
RFM quartile			
Quartile1	Reference	Reference	Reference
Quartile2	-0.026 (-0.033, -0.018)	-0.026 (-0.033, -0.018)	-0.028 (-0.030, -0.026)
Quartile3	-0.057 (-0.064, -0.049)	-0.054 (-0.062, -0.047)	-0.050 (-0.052, -0.047)
Quartile4	-0.083 (-0.091, -0.076)	-0.080 (-0.088, -0.073)	-0.079 (-0.082, -0.076)
P for trend	P < 0.001	P < 0.001	P < 0.001

**Table 3:** Saturation effect curves of RFM and Lumbar BMD.

RFM	Adjusted β (95% CI) P value
Fitting by linear regression model	-0.004 (-0.005, -0.004) <0.0001
Fitting by two-piecewise linear regression model	
Inflection point	20.097
<20.097	-0.002 (-0.003, -0.001) <0.0001
>20.097	-0.005 (-0.005, -0.004) <0.0001
Log likelihood ratio test	<0.001

### 3.3 ROC analysis

**Fig 3:**
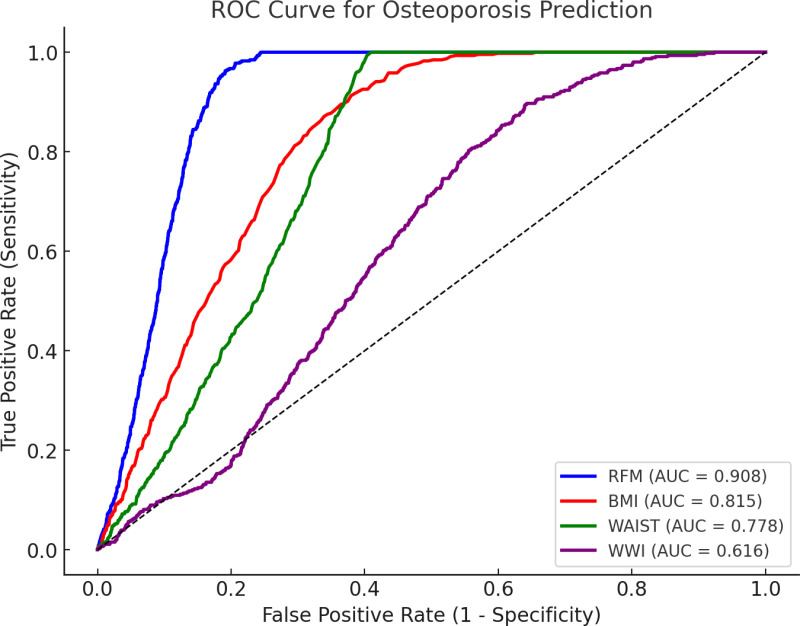
ROC curve of RFM, BMI, WWI and WC.

We conducted a ROC analysis of the diagnostic capabilities of RFM, BMI, WWI and WC for OP. RFM was found to be a better predictor of OP than BMI and WC (Fig 3 and Table 4).

**Table 4:** Results of ROC analysis.

Test	ROC area(AUC)	95%CI (low)	95%CI (upp)	Best threshold	Specificity	Sensitivity	P-value
RFM	0.908	0.902	0.915	42.045	0.794	0.978	Reference
BMI	0.815	0.802	0.828	29.450	0.620	0.913	<0.001
WC	0.778	0.766	0.791	98.550	0.590	1.000	<0.001
WWI	0.616	0.597	0.635	10.896	0.357	0.897	<0.001

### 3.4 Subgroup analyses

When subgroup analyses were undertaken, RFM was found to be inversely linked with Lumbar BMD in both male and female populations after fully accounting for modeling. Males (β= -0.005, 95% CI: -0.005, -0.004) and females (β= -0.004, 95% CI: -0.004, -0.004) had a significant interaction (P < 0.0001). We can see that as RFM increases, the drop in Lumbar BMD is larger in the male population. RFM was also inversely linked with Lumbar BMD in all subgroups, including gender, race, education level, moderate recreational activities, hypertension, diabetes, and smoking status (Table 5).

**Table 5:** Results of subgroup analyses.

Subgroup	β (95%CI)	P-value	P for interaction
Gender			<0.0001
Male	-0.005 (-0.005, -0.004)	<0.0001	
Female	-0.004 (-0.004, -0.004)	<0.0001	
Race			0.0998
Mexican American	-0.005 (-0.005, -0.004)	<0.0001	
Other Hispanic	-0.005 (-0.005, -0.004)	<0.0001	
Non-Hispanic White	-0.004 (-0.005, -0.004)	<0.0001	
Non-Hispanic Black	-0.004 (-0.005, -0.004)	<0.0001	
Other Race	-0.004 (-0.005, -0.004)	<0.0001	
Education level, (%)			0.0454
Less than 9th grade	-0.005 (-0.006, -0.005)	<0.0001	
9-11th grade (Includes 12th grade with no diploma)	-0.005 (-0.005, -0.004)	<0.0001	
High school graduate/GED or equivalent	-0.004 (-0.005, -0.004)	<0.0001	
Some college or AA degree	-0.004 (-0.005, -0.004)	<0.0001	
College graduate or above	-0.004 (-0.004, -0.004)	<0.0001	
Moderate activities			<0.0001
Yes	-0.004 (-0.004, -0.004)	<0.0001	
No	-0.005 (-0.005, -0.004)	<0.0001	
hypertension			<0.0001
Yes	-0.003 (-0.003, -0.002)	<0.0001	
No	-0.005 (-0.005, -0.005)	<0.0001	
Diabetes			<0.0001
Yes	-0.002 (-0.002, -0.001)	<0.0001	
No	-0.005 (-0.005, -0.004)	<0.0001	
Smoking			<0.0001
Yes	-0.004 (-0.004, -0.003)	<0.0001	
No	-0.005 (-0.005, -0.005)	<0.0001	

## 4. Discussion

This is the first study to examine the relationship between RFM and BMD. We found a significant negative correlation between RFM and lumbar BMD. Based on subgroup analyses and interaction tests, this negative correlation was observed for all subgroups except race. ROC analyses showed that RFM had higher sensitivity for detecting OP compared to BMI, WC, and WWI. It also demonstrates the necessity of regulating body fat status for BMD.

The relationship between obesity and OP remains controversial. Some studies have shown that obesity prevents OP [18,24,25]. The “obesity paradox” has also been proposed to explain this phenomenon [26,27]. In previous studies, BMI and WC have often been used to investigate the association between obesity and BMD, but the results were inconsistent. Some studies have shown that an increase in BMI within a certain range can lead to an increase in skeletal muscle mass and fat mass [28]. Individuals with higher muscle mass tend to have higher BMD [29,30]. Moderate weight gain may also increases mechanical load on bones, which stimulates bone formation and improves BMD [31,32]. However, excessive fat accumulation has been associated with lower BMD [33,34].

In a cross-sectional study of 5337 participants, Liu H discovered a positive association between BMI and multi-site BMD (p < 0.001) [35]. Similarly, a cross-sectional research of 2903 adults over 50 years old reported a positive relationship between BMI, WC, and BMD [23]. A meta-analysis indicated that BMI was positively associated with BMD and negatively associated with OP in obese individuals [25]. However, some studies have presented conflicting evidence. A higher BMI is associated with an increased risk of periprosthetic hip fracture [36]. In addition, excessive bone marrow fat in obese individuals may negatively affect BMD, and obesity-related inflammation could further impact bone mass [37,38]. In a clinical research involving 1013 women, WC was found to be negatively linked with BMD [39]. Data from the 4th Korean National Health and Nutrition Examination Survey, WC was negatively related with femoral neck and spinal BMD in both male and postmenopausal female populations [40].

In recent years, research on the relationship between adiposity and BMD has increased. Evidence suggests that body composition plays a crucial role in bone health. However, the effects of different body components on BMD remain controversial, particularly the association between fat mass (FM) and BMD, which lacks consistent conclusions. Studies have examined the impact of lean mass (LM) and FM on BMD and found that both contribute, but LM has a stronger effect. In Korean men, both LM and FM were significant determinants of BMD, whereas in women, only LM was significantly associated with lumbar BMD [41]. Similarly, a study on postmenopausal Vietnamese women found that both LM and FM were independent predictors of BMD, but FM had a weaker effect, emphasizing the key role of LM in bone health [42]. Additionally, research on non-obese postmenopausal Chinese women reported that FM was positively associated with BMD, while central fat accumulation had a negative impact, highlighting the differential effects of fat distribution on BMD [43]. Recent studies have also explored new obesity measures, such as the WWI, which has been negatively correlated with total BMD in adolescents [13]. These findings suggest the need for novel approaches to better understand the relationship between fat distribution and BMD. In measuring the impact of adiposity on health, RFM as an obesity index has received increasing attention in recent years. RFM assesses the percentage of total body fat, is simple to calculate, and is gender-specific [44]. A prospective study found that the risk ratio for all-cause and cardiovascular mortality increased sharply when RFM exceeded 30 in men or 45 in women [45]. RFM has been shown to have a stronger association with depression than BMI and WC [46]. In our ROC curve analysis, RFM demonstrated a higher ability to diagnose OP compared to WWI, BMI, and WC (P < 0.001). These results highlight the importance of RFM in predicting disease as an indicator of obesity. Compared with previous studies, RFM allows for a more comprehensive assessment of individual fat distribution and is more suitable as a predictor of OP.

Numerous studies on adults and adolescents have shown that body fat-based definitions of overweight and obesity can serve as diagnostic tools [44,47–49]. In our study, RFM was significantly and inversely correlated with lumbar BMD. This result helps us to study related diseases such as low BMD [50,51]. Saturation effect data were also shown for K < 20.097 (β= -0.002, 95% CI: -0.003, -0.001) and for K > 20.097 (β= -0.005, 95% CI: -0.005, -0.004). These results suggest that as RFM exceeds 20.097, the negative correlation between RFM and lumbar BMD becomes more pronounced. One possible explanation is that RFM, which incorporates WC, reflects abdominal fat levels. Previous studies have reported a negative association between BMD and abdominal fat. An 8-year follow-up study found that individuals with lower BMI but higher abdominal fat had a significantly increased risk of hip fracture [52].

According to previous studies, gender and racial differences may influence RFM and BMD [53–55]. In our study, the inverse relationship between RFM and lumbar BMD was stronger in men than in women. This may be due to the different patterns of fat distribution in men and women. Women usually have more fat in the hips and thighs, while men have more in the abdomen [56,57]. Additionally, differences in weight and hormone levels between men and women may also affect this relationship [58,59]. Individuals with type II diabetes may have a lower risk of OP and a positive association with BMD [60,61]. A meta-analysis of 39,491 participants revealed a negative correlation between hypertension and BMD [62]. This tends to indicate that there are complicated and variable effects on BMD in populations with diabetes and hypertension. However, when we used RFM to assess lumbar BMD across different populations, we found minimal differences. This implies that compared to BMI and WC, RFM may be a more reliable and generalizable measure for evaluating BMD.

The relationship between obesity and OP has gained increasing attention in recent years. Adipose tissue releases cytokines, such as TNF-α and IL-6, which can disrupt bone metabolic homeostasis [63,64]. Ohori *et al*. found that inhibiting TNF-α through IL-33 reduced bone resorption [65]. TNF-α was also found to be associated with bone loss in an obese mouse model [66]. He B *et al*. reduced the number of TRAP-positive multinucleated osteoclasts by inhibiting IL-6 [67]. Oxidative stress is more likely to occur in obese populations and the mediators it produces can promote osteoporosis [68]. The catalytic subunit of NADPH oxidase, NOX2, has been shown to produce high levels of reactive oxygen species (ROS) in obese mice. Knockdown of NOX2 led to a reduction in the pro-osteoclastogenic cytokines RANKL and histone K [69]. Bone marrow stromal cells (MSC) in high-fat-fed mice showed inhibition of osteogenic differentiation in vitro, while reduced levels of alkaline phosphatase and collagen were also found [70].

Although we found a negative correlation between RFM and lumbar BMD in a large-sample database, this study has limitations. Notably, causality cannot be determined in cross-sectional studies. Due to sample size limitations, we were unable to predict more ethnicities and populations from different countries for analysis. ROC analyses were performed primarily to compare the diagnostic power of different indices and no predictive modelling was performed. The database was unable to find data on femoral intertrochanteric region and femoral neck BMD in 2011–2012 and 2015–2016. Future inclusion of additional databases and in-depth studies are needed to elucidate the relationship between RFM and BMD.

## 5. Conclusion

In conclusion, we discovered a negative association between RFM and Lumbar BMD in US adults. This finding emphasises the feasibility of RFM as an assessment tool for low BMD. However, further studies are needed to prove it.
